# IrGO: Iranian traditional medicine General Ontology and knowledge base

**DOI:** 10.1186/s13326-021-00237-1

**Published:** 2021-04-16

**Authors:** Ayeh Naghizadeh, Mahdi Salamat, Donya Hamzeian, Shaghayegh Akbari, Hossein Rezaeizadeh, Mahdi Alizadeh Vaghasloo, Reza Karbalaei, Mehdi Mirzaie, Mehrdad Karimi, Mohieddin Jafari

**Affiliations:** 1 Department of Traditional Medicine, School of Persian Medicine, Tehran University of Medical Sciences, Tehran, Iran; 2 Department of Biology, Temple University, PA, USA; 3 Department of Applied Mathematics, Faculty of Mathematical Sciences, Tarbiat Modarres University, Jalal Ale Ahmad Highway, Tehran, Iran

**Keywords:** Iranian traditional medicine, Persian medicine, Unani medicine, Ontology, Knowledge base, Mizaj, Herbal medicine

## Abstract

**Background:**

Iranian traditional medicine, also known as Persian Medicine, is a holistic school of medicine with a long prolific history. It describes numerous concepts and the relationships between them. However, no unified language system has been proposed for the concepts of this medicine up to the present time. Considering the extensive terminology in the numerous textbooks written by the scholars over centuries, comprehending the totality of concepts is obviously a very challenging task. To resolve this issue, overcome the obstacles, and code the concepts in a reusable manner, constructing an ontology of the concepts of Iranian traditional medicine seems a necessity.

**Construction and content:**

*Makhzan al-Advieh,* an encyclopedia of *materia medica* compiled by Mohammad Hossein Aghili Khorasani, was selected as the resource to create an ontology of the concepts used to describe medicinal substances. The steps followed to accomplish this task included (1) compiling the list of classes via examination of textbooks, and text mining the resource followed by manual review to ensure comprehensiveness of extracted terms; (2) arranging the classes in a taxonomy; (3) determining object and data properties; (4) specifying annotation properties including ID, labels (English and Persian), alternative terms, and definitions (English and Persian); (5) ontology evaluation. The ontology was created using Protégé with adherence to the principles of ontology development provided by the Open Biological and Biomedical Ontology (OBO) foundry.

**Utility and discussion:**

The ontology was finalized with inclusion of 3521 classes, 15 properties, and 20,903 axioms in the Iranian traditional medicine General Ontology (IrGO) database, freely available at http://ir-go.net/. An indented list and an interactive graph view using WebVOWL were used to visualize the ontology. All classes were linked to their instances in UNaProd database to create a knowledge base of ITM materia medica.

**Conclusion:**

We constructed an ontology-based knowledge base of ITM concepts in the domain of *materia medica* to help offer a shared and common understanding of this concept, enable reuse of the knowledge, and make the assumptions explicit. This ontology will aid Persian medicine practitioners in clinical decision-making to select drugs. Extending IrGO will bridge the gap between traditional and conventional schools of medicine, helping guide future research in the process of drug discovery.

## Background

Iranian traditional medicine (ITM), also known as Persian medicine (PM) and familiar to some as Unani medicine, is a holistic school of medicine, founded on a philosophical theory with a categorical framework. The body of collected knowledge in ITM has been extensively documented and revised throughout the centuries. Persian scholars preserved a large amount of the Greco-Roman medicinal application of plants while eventually complementing and expanding it with their domestic knowledge and experiences [1]. The scientific basis of ITM, evident through many examples including *Avicenna’s Canon of Medicine*, being a primary medical reference in Europe until the sixteenth century [2], make this traditional school of medicine a powerful and innovative source for acquisition of data. Persian scholars have used extensive terminology to describe concepts of this medical school. However, the numerous concepts are, at times, not explicit, both in terms of definition and in the complex relationships between them. Coupled with a large amount of data collected through thousands of years, this ambiguity impedes the acquisition of knowledge from this precious resource.

To address this issue and code the concepts in a reusable manner, constructing an ontology of the concepts of ITM seems a necessity. An ontology is a formal explicit description of a domain, consisting of taxonomy as the backbone, universals and defined classes, and the relationships that exist among them [3]. It serves to represent the structure of a domain in order to encode specific information about the theories and preliminary principles of that science and elucidate the implicit laws. The semantic network of an ontology consists of nodes that represent entities, and edges that represent the semantic relationships that exist between the concepts.

Such representation will eliminate ambiguities in concepts and provide an integrated terminology in this domain. As a result, an understanding of the natural language of ITM by intelligent systems, analysis of the relationships between concepts, and extraction of hidden relationships will be made possible. It will serve to integrate data across heterogeneous databases [4]. Moreover, the accuracy of clinical diagnosis and decision-making in treatment can be enhanced through building an ontology and knowledge-base of ITM concepts.

Over the last decades, ontologies have become prevalent in biomedicine [5]. An example in the biomedical domain includes Gene Ontology (GO) [6], used as a means to standardize terminology, to enable access to domain knowledge, to verify data consistency and to facilitate integrative analyses over heterogeneous biomedical data [4]. Moreover, there is a trend towards standardizing traditional medical systems by creating semantic networks and ontologies. One of the most comprehensive works in this regard is the traditional Chinese Medicine Language System (TCMLS), with over 120,000 concepts, 300,000 terms, and 1.27 million semantic relational links [7]. TCMLS is an extensive semantic network and thesaurus, which has recently been used to create a middle-level ontology of TCM concepts to strengthen the foundations for natural language processing, semantic retrieval, clinical decision making in traditional Chinese medicine [8].

No study has yet been conducted to create a language system or ontology of ITM concepts to the best of our knowledge. A major domain of any medical system is *materia medica*, i.e., the body of collected knowledge about the therapeutic properties of any substance used for healing. Furthermore, one of the most critical treatment principles in ITM is taking into account the *Mizaj* (temperament) [9] of patients, diseases, and drugs [10, 11]. Other features of medicinal substances including their actions, the diseases and the organs they are effective on, their adverse effects, and dosage are also important parts of treatment strategies in ITM. Thus, creating an ontology and knowledge-base of the features of medicinal substances as the first step in formal representation of ITM concepts seems relevant. Besides aiding ITM physicians in research and decision making, it will also be an aid in new drug discovery. Utilizing the ethnomedical use of plants based on traditional medical systems is a starting point to discover new drugs [12, 13], which can be more effectively carried out once the information from these resources has been standardized and systematized.

This research was designed to systematically collect the concepts used to describe ITM *materia medica* regarding their main features in order to create an ontology and subsequently, an ontology-based knowledge base of *materia medica*.

## Construction and content

### Domain and scope

One of the most important domains in any medical system is the treatment modalities. In the case of traditional medicines, a chief element of treatment is the use of natural medicinal substances, including herbs, minerals, and animal-derived drugs. IrGO is intended to represent the concepts used to describe *materia medica* in ITM literature. As described, these include Mizaj, drug actions, effects on diseases and body organs, substitute, corrigent, and dosage.

### Resource

To make the ontology both comprehensive and accomplishable, a single resource was selected according to the criteria of being the most recent, containing the largest number of monographs, and being compiled by a prominent ITM scholar [14]. *Makhzan al-Advieh* (Treasury of drugs)*,* composed by Mohammad Hossein Aghili Khorasani, a renowned physician and pharmacologist of the eighteenth century, was selected as the resource for this project. One of the best and most comprehensive resources of ITM, this semi-structured encyclopedia includes 1741 principal herbal, animal, and mineral preliminary monographs. They are described by their identity, Mizaj, actions and medicinal uses, dosage, adverse effects, substitutes, and corrigents. In a recent project, descriptions of the medicinal substances in Makhzan al-Advieh along with their scientific names and common names have been gathered in a database called UnaProd (Universal Natural Product Database) available at http://unaprod.com [15].

### Ontology creation methods

IrGO was developed according to principles of the Open Biological and Biomedical Ontologies (OBO) Foundry (http://obofoundry.github.io/principles/fp-000-summary.html), using Protégé 5.5.0, which supports the latest Web Ontology Language (OWL) specification and is the most extensively used editor for development and management of ontologies [16].

To develop the ontology, a number of steps were followed. The first task included compiling a list of classes for each part of the ontology including Mizaj, actions, organs, and diseases. Initially, classes for each main part were extracted via text mining of the resource and examining reference texts*,* followed by manual reviews based on results to create a finalized taxonomy of classes. Moreover, Avicenna’s *Canon of Medicine* [17] and the preliminary chapters of *Makhzan al-Advieh* (explaining term definitions, estimation of drug properties, instructions on drug preparations) were studied as an aid in classifying concepts.

The classes were then arranged in a taxonomy, which is a hierarchy consisting of the concepts linked by sub-type relations. The selected method to construct the taxonomy was a combination of top-down and bottom-up approaches. An initial approach was to use a top-down method, as the more general terms are defined and already organized in descriptive form in ITM literature. However, there were also classes in lower levels which were not defined explicitly in textbooks but used to describe monographs in our selected resource. These concepts were studied, and their specifications were determined to be arranged using a bottom-up strategy to complete the model.

Each class was subsequently provided with an Aristotelian definition that takes into account the genus and differentia of terms [3]. In other words, every term in the ontology is provided with a definition, formulated through the specification of how the instances of the universal represented by the relevant term are differentiated from other instances designated by its parent term. This formulation of definitions ensures the correctness of the hierarchy, with each definition taking us back to the root node when unpacked. Moreover, circularity is automatically avoided. Root nodes in each taxonomy were defined based on ITM literature.

The next step consisted of determining data properties, object properties and their cardinalities. Subsequently, annotation properties, including a code (ID), an English label, transliterations, alternative terms (synonyms), and definitions (Persian and English) were determined for concepts. Consistency of IrGO was checked using the HermiT 1.4.3.456 reasoner [18].

## Utility and discussion

### Extraction of IrGO classes

#### Mizaj

The foundational natural philosophy of ITM explains phenomena by the two pairs of opposing qualities, namely hotness-coldness and wetness-dryness. The qualities of hotness and coldness affect wetness and dryness, thus called active qualities, whereas the latter pair are affected by hotness and coldness, thus called passive qualities. The four illustrative elements of fire, wind, water, and earth exhibit specific qualities with that of the fire being hot and dry, the wind hot and wet, the water cold and wet and the earth cold and dry. It is noteworthy that, these elements are symbolic, and not literally, what we perceive of them in the physical world. They are indivisible matter named according to the qualities and potentialities they induce in a substance. For example, a substance containing the element fire is light-weighted and permeable, fluidity and flexibility denote presence of the element water.

Elements constitute the primary components of virtually all matter, living and non-living. When intermixed in various proportions, they act upon and react with one another, ultimately reaching a state of equilibrium and a new quality called *Mizaj* or temperament. As a critical concept of ITM, *Mizaj* is a quality of all beings in the universe. In addition to living and non-living things, a certain *Mizaj* is attributed to variables such as places, climates, and seasons, based on the prediction of the effect they have on the human body. *Materia medica*, whether herbal, animal, or mineral are no exception, the Mizaj of which is determined based on the change of body *Mizaj* in a healthy young adult upon consuming the substance.

Upon entering a body with a balanced Mizaj and actualization of potentials under the influence of metabolic processes, a substance with a balanced Mizaj does not change the principle Mizaj of the body, nor does it lead to any deficit or disturbance in body functions. Unbalanced substances, on the other hand, can change the principle temperament of the body, leading to alterations in body functions. The magnitude of these changes is expressed as degrees in a range from one to four for each of the qualities, each of which is further classified into three degrees of minimum, medium, and maximum. For example, a drug with a hot and dry Mizaj is described via a degree in hotness and a degree in dryness.

As described in ITM literature, ten main Mizaj types in two major classes, namely balanced and unbalanced, have been defined for *materia medica*, with the unbalanced ones as follows: hot, cold, wet, dry, hot-wet, hot-dry, cold-wet, cold-dry, and *Morakkab al-Ghovaa* (a Mizaj ascribed to drugs that can exhibit properties of two or more of the mentioned Mizaj types).

**Fig. 1:**
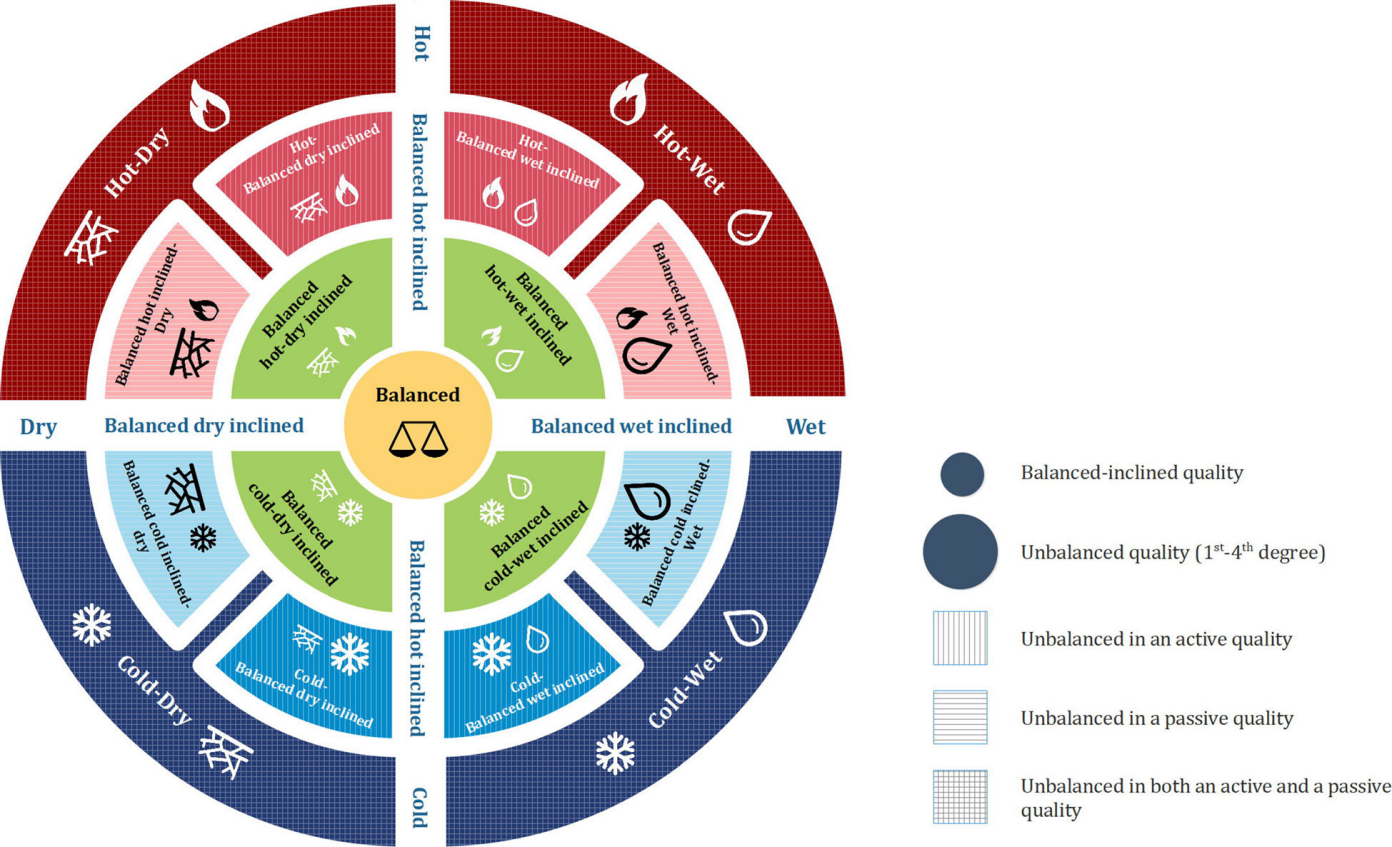
Mizaj types. The vertical axis is an indicator of active qualities with points lower than balanced being cold and higher points being hot. The horizontal axis displays passive qualities, with the dry quality to the left. The nine main mizaj types of ITM include balanced, hot, cold, dry, wet, hot-wet, cold-wet, hot-dry, cold-dry. Other Mizaj types illustrated in the second and third inner circles of the figure are more precise indications of degrees used in descriptions of the Mizaj of drugs in Makhzan al-Advieh with the green zone in the balanced region and the light red/blue zones counting as unbalanced in one active/passive quality. Smaller icons of qualities demonstrate a balanced-inclined (slight) quality while larger ones are indicative of an unbalanced quality, which can lie in a range between first to fourth degree. Mizaj types with horizontal stripes are unbalanced in one of the passive qualities, while the vertically-striped are unbalance in an active quality. The four checkered Mizaj types are unbalanced in both their active and passive quality

Text mining the Mizaj field of drugs revealed that Persian scholars used more precise terms in describing the Mizaj of medicinal substances. These terms mainly pertained to the degree of qualities but also resulted in the necessity to create classes other than the ten main ones (the most inner and outer circles in Fig. 1). Moreover, there were drugs with a balanced Mizaj that were said to have slight degrees of one or two qualities. Examples include “Judas tree” (*Orjovan*), which is balanced though with slight hotness, and “common marshmallow” that has a balanced Mizaj with slight coldness and wetness. Therefore, eight subtypes were created for the balanced class, four with slight degrees in only one quality (demonstrated on the vertical/horizontal axis in Fig. 1), and four with slight degrees in both an active and a passive quality (the green third inner circle in Fig. 1).

Moreover, some drugs had slight degrees in one of the qualities, and a full degree for another. For example, “black gram” has an unbalanced cold Mizaj with slight dryness. As these instances did not fit into either hot or hot-dry Mizaj types, eight further classes were also added to the hierarchy (the second inner circle in Fig. 1).

**Fig. 2:**
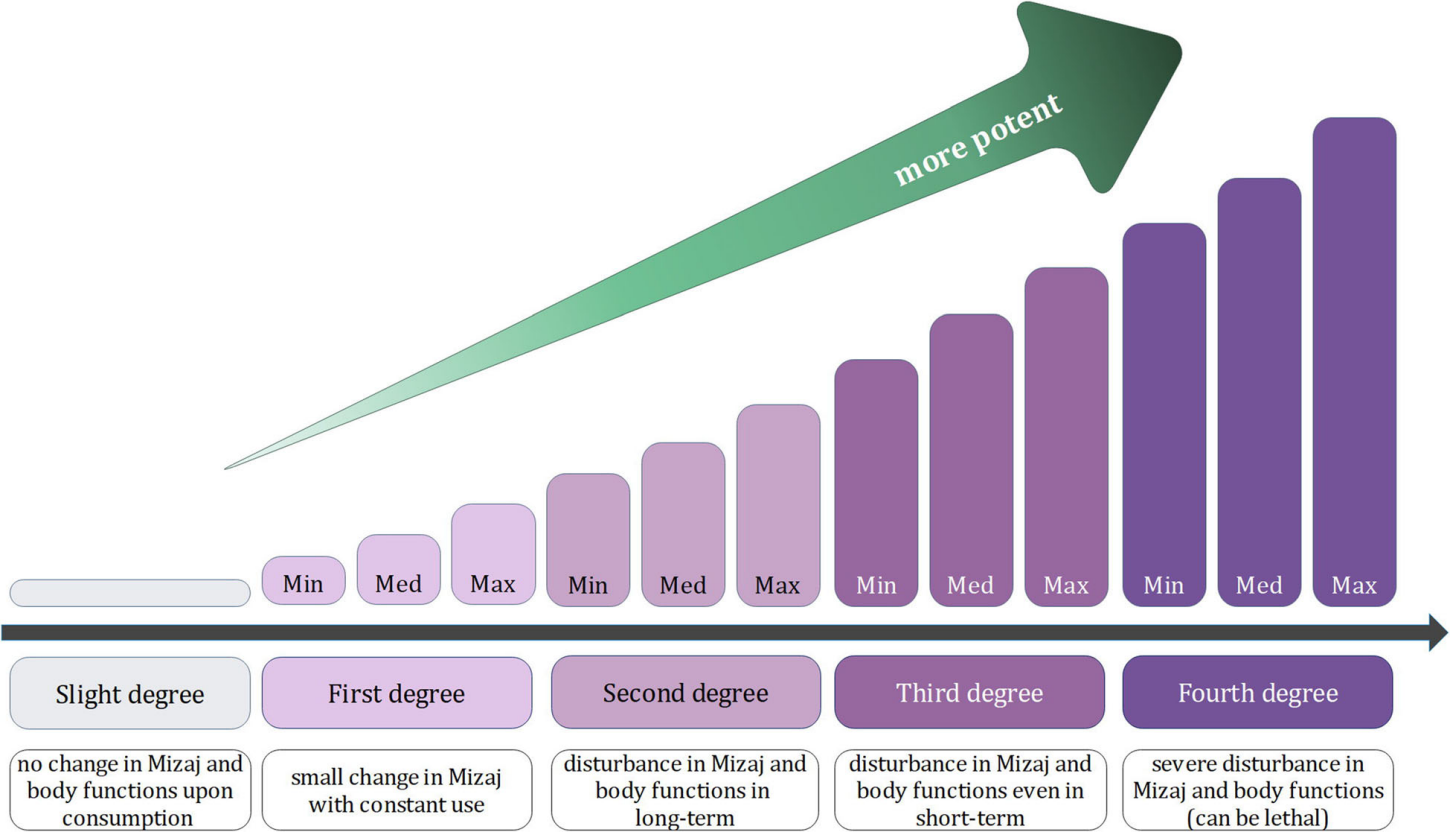
Degrees of qualities. The degrees used to describe each of the four qualities (hotness, coldness, wetness, dryness) range from slight to maximum in fourth degree. This classification based on the potency to induce changes in Mizaj and body functions of a young, healthy person upon consumption

As described above, the quality degrees described for Mizaj are the power of the qualities to impose changes in the body. In ITM literature, these are expressed in four degrees, where each is further classified into minimum, medium, and maximum (Fig. 2). The higher the degree of a drug is, the more potent it would be in changing the Mizaj and body functions. According to the definitions in the introductory chapters of Makhzan al-Advieh, a drug in first degree can change the Mizaj of a person only when consumed in large amounts or over a long time but leads to no disturbance in body functions. Second degree drugs tend to change body Mizaj in small amounts, but again do not result in body dysfunctions even in large amounts or with constant use. Drugs in the third degree can lead to a disturbance in body functions in addition to changing the Mizaj, while fourth degree drugs cause severe disturbance and may be lethal. Based on the terms extracted by text mining, a slight degree was also ascribed to each of the four qualities. This class indicates an inclination of a balanced Mizaj towards one of the qualities that do not reach first degree.

#### Actions

One of the most important attributes of drugs are their actions, which indicate the pharmacological effects of medicinal substances. One of the introductory chapters of *Makhzan al-Advieh* is dedicated to describing 67 main drug actions, along with a rough classification and definitions. The main categories of actions include:

- *universal actions:* actions pertaining directly to one of the four qualities (for example heating);
- *quasi-universal actions:* which are described as actions that influence the whole body similar to universal actions, but exert a specific effect (for example a diuretic induces the specific action of urination, which in turn has an effect on all body systems);
- *particular actions:* actions with specific effects in certain conditions and disorders (for example a sealant drug acts on wounds).

The preliminary list of actions was used for text mining more specific actions in the resource. A list of 5562 phrases were extracted, which were manually reviewed. Distinct phrases were grouped and arranged as subclasses or alternative terms of the original action. Moreover, the list was gradually completed by other actions that were detected in a manual review of the section describing actions for each of the drugs. The list was reviewed multiple times, and enhanced by determining terms that were synonyms of others, and also differentiating terms in homonyms. The resulting list comprised 609 actions of *materia medica*.

#### Medicinal uses (diseases)

The medicinal uses of drugs are in fact instances of diseases, for which drugs are used in prevention, management or cure. Classification of diseases in ITM is organ-based and in many cases symptom-based. Since extracting a list of diseases was not possible via text mining techniques, a preliminary list was obtained from therapeutic books including *Exir-e Azam* (The Great Panacea) [19], *Teb-be Akbari* [20], *Mo’alejat-e Aghili* [21], and Canon of Medicine [17]. This list of 1621 diseases, was completed by text mining the resource and finally a manual review to ensure comprehensiveness. The final list included 2456 disease names along with their alternative terms.

#### Body entities

Regarding body entities, a list of organs were gathered from *Tashrih-e Mansouri* (Mansouri’s Anatomy) [22] and Canon in Medicine [17]. As body substances usually follow an action (for example dissolver of phlegm), these terms were obtained from the list of actions extracted by text mining. A number of terms in *Makhzan al-Advieh* describing body entities did not exist in the lists described (for example body exterior); so, both text mining and a final manual review of the resource was also carried out. The final list of body entities included 282 terms, including 151 organs, 12 body subdivisions, 13 immaterial body entities and 106 body substances.

### Construction of IrGO

As a summary of class extraction described in the previous section, a general categorization of the concepts used to describe materia medica exists in ITM literature. However, text mining and manual review of the resource revealed many further classes, which were included for a comprehensive taxonomy of terms and phrases used to describe the properties and medicinal uses of *materia medica* in ITM.

Following term extraction as described in the previous section, classes of Mizaj, actions, body entities, and diseases (medicinal uses) were arranged in separate taxonomies using a combination of top-down and bottom-up strategies. Each were reviewed and enhanced several times to include all extracted classes in a single inheritance taxonomy, while conforming to ITM categorizations to be a shared view and thus usable by specialists in this domain.

**Fig. 3:**
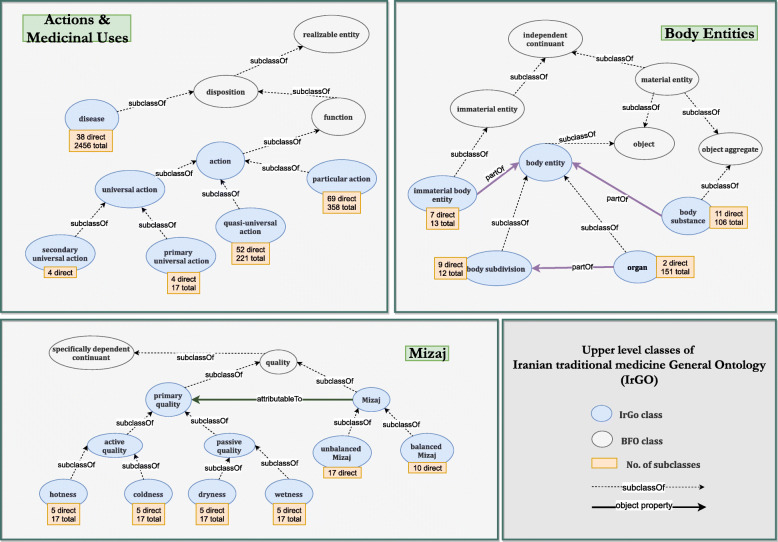
Upper level classes of Iranian traditional medicine general ontology (IrGO) incorporated into Basic Formal Ontology (BFO). The number of direct and total subclasses have been specified for each class

The main classes along with the number of direct and total subclasses for each taxonomy are illustrated in Fig. 3. Classes were annotated with a unique ID (IrGO_‘7-digit-number’), English and Persian labels, alternative terms (when present), transliteration, and English and Persian definitions (currently available for Mizaj and action taxonomies).

**Fig. 4:**
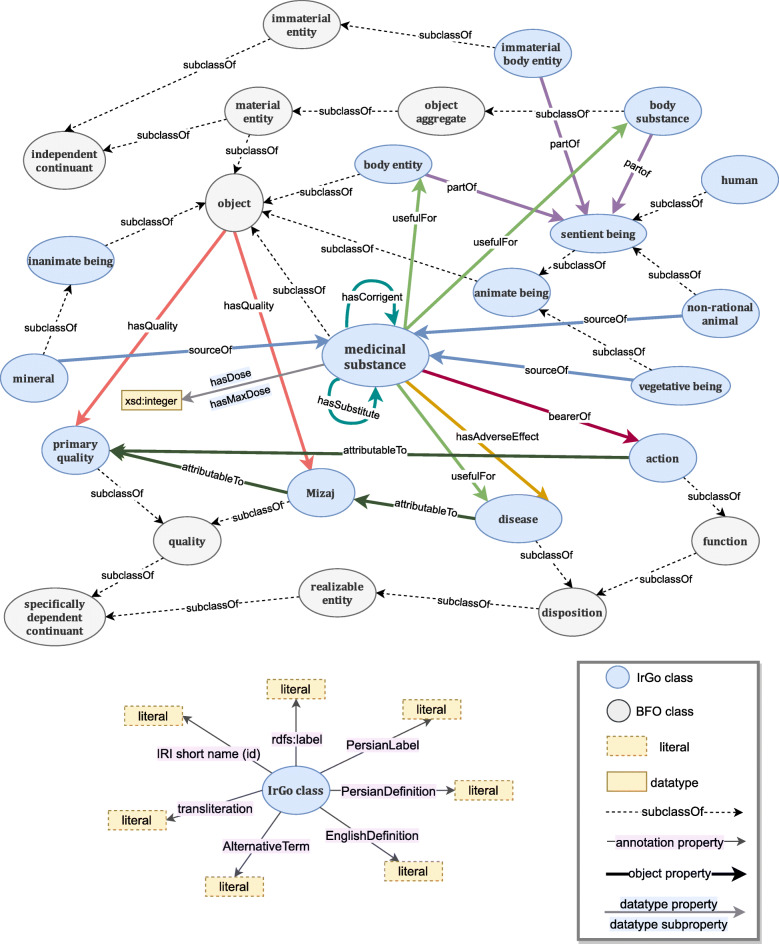
A conspectus of Iranian traditional medicine general ontology (IrGO), illustrating upper classes in taxonomies of Mizaj, action, disease, and body entity along with properties relating them to medicinal substances. The annotation properties provided for IrGO classes are depicted in the bottom diagram

Subsequently, the taxonomies were placed in proper positions in BFO. Mizaj and the primary qualities were considered as a quality (BFO_0000019), which is in turn a subclass of specifically dependent continuant (BFO_0000020). Disease was classified under disposition (BFO_0000016) and actions under function (BFO_0000034), both of which are subclasses of a realizable entity (BFO_0000017). Body entities were categorized into three subclasses: body entities as a subclass of object (BFO_0000030), body substance as a subclass of object aggregate (BFO_0000027), and immaterial body entity as a subclass of immaterial entity (BFO_0000141). Moreover, classes of medicinal substance, animate body and inanimate body, along with their subclasses (demonstrated in Fig. 4) were added to the ontology to enable linking between the concepts.

To finish, object properties and cardinalities were determined. The finalized ontology includes 3521 classes, 15 properties, and 20,903 axioms (Fig. 4).

### IrGO evaluation

Evaluation is an integral part of ontology development process, ensuring applicability and interoperability [23]. A common approach would be to assess different aspects or levels of the ontology independently, since ontologies are complex structures [23]. Generally, ontologies are evaluated for accuracy, consistency, completeness, conciseness, clarity, and computational efficiency [24, 25]. Approaches to ontology evaluation are categorized into four categories of gold-standard-based, criteria-based, task-based, and corpus-Based [25, 26]. IrGO was evaluated using a criteria-based approach to assess the structural properties of the ontology, and a task-based approach as an instance of how the ontology could be applied to retrieve knowledge from the ITM resource used to build the knowledge base.

#### Criteria-based evaluation

Criteria-based approaches include structure-based and complex/expert-based assessments. The structural properties of IrGO were computed using Ontometrics [27]. As presented in Table 1, base metrics, like counting of classes, properties, and axioms (including class axioms, object property axioms, and annotation axioms), have been provided for IrGO as a whole as well as each of the subontologies (Mizaj, action, body entity, and disease).

**Table 1:** Base metrics of IrGO calculated by Ontometrics

Metrics	Mizaj	Action	Body entity	Disease	IrGO
Axiom	1046	4273	1698	13,562	20,903
Logical axiom count	143	614	384	2455	3627
Class count	123	609	289	2435	3521
Object property count					13
Data property count					2
Annotation property count					11
Class axioms					
SubClassOf	131	607	376	2448	3615
Disjoint classes axioms count					4
Object property axioms					
SubObjectPropertyOf axioms count					4
Inverse object properties axioms count					2
Asymmetric object property axioms count					2
Annotation axioms					
Annotation assertion	764	3034	1008	8656	13,741
**Schema Metrics**					
Inheritance richness	1.06	1.00	1.30	1.01	1.02
Relationship richness	0.11	0.00	0.01	0.00	0.01
Axiom/class ratio	8.50	7.01	5.87	5.56	5.94
Calss/relation	0.83	1.00	0.76	0.99	0.97

A number of schema metrics were also calculated (Table 1). Inheritance richness shows the distribution of information across different levels of ontology. For IrGO, this is not a high number (1.02). This shows that the ontology is deep (vertical), indicating that it has covered the domain in detail. The highest inheritance richness belongs to body entity, which is indicative of this subontology being horizontal, where classes have the largest number of direct subclasses compared to other subontologies of IrGO.

Relationship richness is indicative of the types of relations in an ontology. This metric is also a low number for IrGO (0.01). It shows most relations in IrGO are inheritance relationships, which is partly due to the large number of classes in taxonomies. The highest level of relationship richness is seen in the Mizaj subontology.

The axiom/class ratio is the average number of axioms per class (5.94). The highest ratios belong to Mizaj and action, as the classes in these two subontologies are provided with both English and Persian definitions. Finally, Class/relation ratio is a metric that describes the ratio between classes and relations (0.97). This ratio was highest for actions and lowest for body entity.

#### Task-based evaluation

A task-based approach was undertaken to assess relations between subontologies of IrGO. According to Persian scholars, Mizaj and the inherent qualities in a medicinal substance are one of the determinants of its actions, and medicinal uses. Thus, based on ITM, these drug characteristics are all connected to each other. To accomplish the evaluation, the actions of drugs with “cold dry Mizaj” (the subset with coldness and dryness both in third degree) were selected as a case study.

**Fig. 5:**
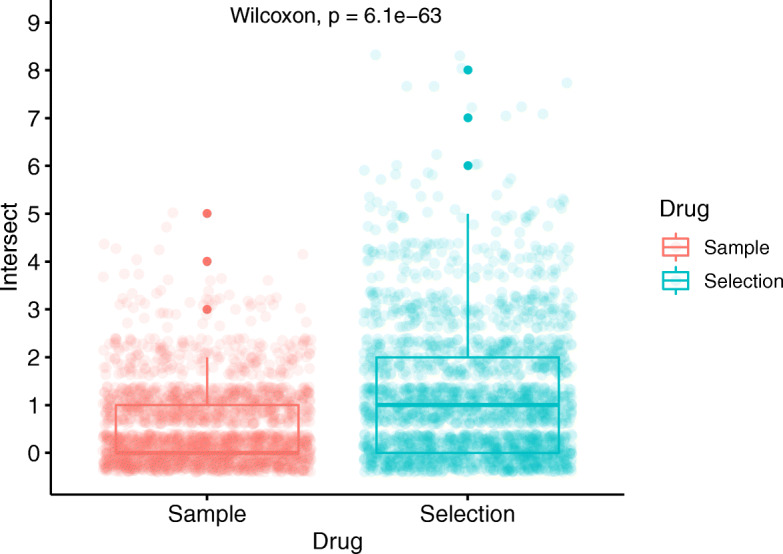
Pairwise calculation of intersect actions between drugs with cold dry Mizaj (coldness and dryness both in third degree) and sample drugs. Mean intersect for each group (1.18 for selection versus 0.52 for sample) is demonstrated as a dashed line

A total of 48 medicinal substances with this feature existed in our knowledge base. Pairwise intersect of actions were calculated for these drugs and also a random sample of 48 other drugs not having this Mizaj type and quality degrees. Mean number of intersects (common actions) in the selection group was 1.18, while it was 0.52 for sample drugs. Also, the mean number of zeros (drug pairs with no intersections) was 19.21 for selected drugs, and much higher (31.88) for samples (Fig. 5). Results of Wilcoxon rank sum test with continuity correction demonstrated that the pairwise intersects calculated for selection and sample were not identical (*p*-value = 6.1e-63).

### Ontology visualization

There are a number of methods to visualize ontologies. A suitable way should support the presentation of ontology ingredients, i.e., classes, relations, instances, and properties, and allow the user to discern information without effort [28]. The Visual Notation specifies a visual language for ontologies represented in OWL for OWL Ontologies (VOWL). Two common ontology visualization techniques are the indented trees and directed acyclic graphs [29]. The indented tree was provided as an organized hierarchical view of IrGO. Also, the graph visualization, which is more controllable and intuitive was presented for the users. We created an interactive graph view using WebVOWL [30] to allow exploration and customization of ontology.

### Web Interface

IrGO was built using PHP 7.2.11 for server-side data processing, and JavaScript ECMAScript 2015. Data was stored in MySQL 10.1.37. An outline and user manual of the database, together with network and indented list of the ontology, are available on the website. The query of database contents by one or more keywords is possible. Classes are linked to their instances in UNaProd database to create a knowledge-base of materia medica of Persian medicine [15].

## Conclusion

We constructed an ontology-based knowledge base of ITM concepts in the domain of *materia medica* to standardize and clearly demarcate concepts in this regard. This was accomplished according to the principles provided by the OBO foundry using Protégé 5.5.0.

Systematizing ITM will reinforce the basis for natural language processing, enable semantic retrieval from the rich sources of this medical school, and help physicians of this field in better clinical decision making. It also has implications in modern medical research, including ethnopharmacology and new drug discovery. Identifying potential new medicines is a process that can be fulfilled through various methods including random (biodiversity-based) [31], chemo-systematic [32], ecological [33], computational [34], and ethnopharmacological [35] approaches. Compared with the random model, utilizing ethnomedical use of plants method is time and money-saving and has a higher hit-rate [13, 36].

Several studies have been dedicated to investigating the attributes of medicinal substances in ITM resources and current data on chemical compounds of drugs. For example, a strong relationship has been demonstrated between the Mizaj of drugs in ITM with their major chemical compounds [37]. Molecular properties of herbal compounds have recently been studies by machine learning approaches and found to have associations with meridians in TCM, providing a deeper insight and also rationales for classifications of this traditional medical system [38, 39]. It seems that the knowledge and experience provided by ITM can help reduce the expenses and time consumed in the process of developing new drugs from natural sources. The efficacy of this method would be even higher in a more organized and standardized model of traditional medicine. Accordingly, IrGO can be used to investigate similarities in chemical compounds, properties of medicinal substances, and the links to associated biological processes and diseases.

In conclusion, developing an ontology-based knowledge-base of ITM concepts will provide a whole range of benefits, including offering a shared and common understanding of ITM, enabling reuse of the knowledge in this field, making the assumptions explicit and finally gaining new knowledge by analyzing the concepts and their relationships. It will also bridge the gap between traditional and conventional schools of medicine, which will, in turn, help guide future research on novel treatment options based on the obtained knowledge.

## Data Availability

Project name: IrGO. Project home page: http://ir-go.net/ Archived version: 1.1.0. Operating system(s): Platform independent. Programming language: R, PHP, JavaScript, JQuery, HTML, CSS. Other requirements: None. License: MPL-2.0.

## Abbreviations

ITM: Iranian traditional medicine
CAM: Complementary and alternative medicine
TCM: Traditional Chinese Medicine
IPA: International phonetic alphabets
IrGO: Iranian traditional medicine General Ontology
UNaProd: Universal Natural Product Database
PM: Persian Medicine
